# Natural history and cycle threshold values analysis of COVID-19 in Xiamen City, China

**DOI:** 10.1016/j.idm.2022.07.007

**Published:** 2022-08-09

**Authors:** Bin Deng, Weikang Liu, Zhinan Guo, Li Luo, Tianlong Yang, Jiefeng Huang, Buasiyamu Abudunaibi, Yidun Zhang, Xue Ouyang, Demeng Wang, Chenghao Su, Tianmu Chen

**Affiliations:** a State Key Laboratory of Molecular Vaccinology and Molecular Diagnostics, School of Public Health, Xiamen University, Xiamen City, Fujian Province, People's Republic of China; b Xiamen Center for Disease Control and Prevention, Xiamen City, Fujian Province, People's Republic of China; c Zhongshan Hospital, Fudan University (Xiamen Branch), Xiamen City, Fujian Province, People's Republic of China

**Keywords:** COVID-19, Natural history, Cycle threshold value

## Abstract

**Objective:**

This study elaborated the natural history parameters of Delta variant, explored the differences in detection cycle thresholds (Ct) among cases.

**Methods:**

Natural history parameters were calculated based on the different onset time and exposure time of the cases. Intergenerational relationships between generations of cases were calculated. Differences in Ct values of cases by gender, age, and mode of detection were analyzed statistically to assess the detoxification capacity of cases.

**Results:**

The median incubation period was 4 days; the detection time for cases decreased from 25 to 7 h as the outbreak continued. The average generation time (GT), time interval between transmission generations (TG) and serial interval (SI) were 3.6 ± 2.6 days, 1.67 ± 2.11 days and 1.7 ± 3.0 days. Among the Ct values, we found little differences in testing across companies, but there were some differences in the gender of detected genes. The Ct values continuous to decreased with age, but increased when the age was greater than 60.

**Conclusion:**

This epidemic was started from aggregation of factories. It is more reasonable to use SI to calculate the effective reproduction number and the time-varying reproduction number. And the analysis of Ct values can improve the positive detection rate and improve prevention and control measures.

## 1 Introduction

Since December 2019, COVID-19 has been a pandemic that causes a serious economic and health burden, with the rapid mutation of SARS-CoV-2, it makes the prevention and control of COVID-19 a constant battle (Jo et al., 2020; Niu et al., 2021). The natural history of a disease is the entire course of the disease from its onset, progression and outcome without any treatment or intervention (Machhi et al., 2020). The natural history of diseases varies widely, and understanding the natural history of a disease is important for early diagnosis and prevention, as well as for determining the effectiveness of treatment (Gautret et al., 2020). The natural history of a disease are consists of four main periods: the susceptible period, the subclinical period, the clinical period, the recovery period, and the disability or death period (Shi, 2007). On September 12, 2021, the first factory-gathering outbreak caused by Delta variant of COVID-19 in China was reported in Xiamen. The outbreak lasted for 21 days, and a total of 236 confirmed cases were reported, affecting 5 administrative districts in Xiamen, mainly in Tong'an Industrial Park, accounting for 88.13% of the total cases. The epidemiological process of an infectious diseases is the process by which an infectious disease occurs, spreads and terminates in a population (Shi, 2007). An epidemic of an infectious disease must contain three basic components: the source of infection, the route of transmission and the susceptible population. These three parts must exist at the same time to constitute an epidemic of an infectious disease. The intergenerational relationship of infectious diseases is of great significance for simulation of transmission dynamics of infectious disease and the prevention as well as control of infectious diseases in the field (Zhao, 2020). There are three time points in natural history that are crucial for calculating intergenerational relationships: time of infection, time of transmission, and the time of symptoms. According to these three-time points, three key indicators of intergenerational relationship can be calculated: Generation time (GT): refers to the time interval between the source of infection and the infection of secondary cases; Time interval between transmission generations (TG): refers to the time interval between the source of infection and the occurrence of contagiousness in secondary cases; Serial interval (SI): refers to the time interval between the source of infection and the onset of symptoms in a secondary case (Zhao, 2020). Epidemiological parameters of COVID-19 transmission dynamics are essential for public health departments to understand case generation turnover and disease transmissibility when implementing interventions. Globally, several studies have been conducted to estimate the mean consecutive interval and incubation period of COVID-19. Some studies found a mean intergenerational time of symptoms of 7–9 days, but the number of cases in the dataset they applied was poorly representative with only a few confirmed cases (Li et al., 2020; Nishiura et al., 2020).

The cycle threshold (Ct) in reverse transcriptase-polymerase chain reaction (RT-PCR) has been used to measure the amount of amplification required for a target viral gene to cross the threshold and is inversely proportional to viral load (Pujadas et al., 2020). Although, SARS-CoV-2 infection may be asymptomatic or symptomatic, and viral load does not correlate to disease severity, detection of viral load is important to prevent the spread of a potential infection (Tom & Mina, 2020). Some studies have shown that the viral load peaks around the onset of severe cases of COVID-19, while mild and moderate cases are infectious for 10 days (Singanayagam et al., 2020). Other studies will also use Ct value as one of the criteria for hospitalization (Seeni et al., 2021). Studies addressing cycle thresholds can even predict disease severity, survival, and 6-month sequelae in patients with COVID-19 and have symptoms (Garg et al., 2021; Trunfio et al., 2021). In this study, the data of the first factory clustered outbreak caused by the Delta variant in China were collected, and the natural history of the Delta variant was described by analyzing the three-basic components of the epidemic. The intergenerational relationship of COVID-19 was used to estimate the transmission rate of the outbreak in the factory, providing theoretical basis and evidential support for more subsequent outbreaks. In addition, this study collected the positive Ct values for nucleic acid screening in confirmed cases and analyzed the relationship between the viral loads of confirmed patients under different conditions in this outbreak.

## 2 Methods

### 2.1 Data collection

We established a COVID-19 outbreak dataset of Xiamen in September 2021 using the epidemic-related data collected by the Xiamen Center for Disease Control and Prevention. The dataset mainly included case-related information, such as gender, age and occupation; it also included the onset time, detection time, and reporting time of all cases. In addition, we collected the cycle thresholds of real-time reverse transcription polymerase chain reaction at the time of first detection and positive retest for most cases in this outbreak.

### 2.2 Calculation of natural history parameters

The incubation period is the gap between the pathogen invades the body and the body reacts or begins to show symptoms. Confirmed cases with clear associated cases and only one single exposure is selected. Incubation period was calculated by the following formula: Incubation period = onset date - single exposure date. Onset-reporting time refers to the time it takes for a case to be reported after onset and can reflect the ability for early detection. The calculation formula is as follows: onset-reporting duration = reporting date - onset date. The duration of diagnosis refers to the time required from the time of nucleic acid testing of a case to the reporting of the test results of a case, which is calculated as follows: Diagnosis duration = reporting date - nucleic acid detection date.

### 2.3 Analysis of intergenerational relationships

The calculation of the generations of infection is based on the duration of infection between two generations of cases, and its calculation relies on a rigorous exposure history investigation. However, in the actual contact mode, effective contact between individuals is affected by the contact method, frequency, time, etc. It is difficult to determine which contact caused the transmission. In this analysis, the interval of GT were calculated as follows: the lower limit of GT = the first contact time of the second-generation cases - the first contact time of the first-generation cases; the upper limit of GT = the last contact time of the second-generation cases - the last contact time of the first-generation cases. To calculate the TG, the time of infection for both generations of cases must be determined. Therefore, the TG for this outbreak was calculated as follows: TG = time of first positive screening for second-generation cases - time of positive screening for first-generation cases; SI of symptoms was calculated in conjunction with the time of onset. A total of 106 people in this outbreak were included to calculate the SI of symptoms. The calculation formula is as follows: SI = onset time of second-generation cases - onset time of first-generation cases.

### 2.4 Cycle threshold values analysis

By analyzing 236 confirmed cases with different detection reagents, genders, ages, etc. in Xiamen. ANOVA, *t*-test, Wilcoxon rank sum test and Kruskal-Wallis test were used to discriminate the differences in viral load of confirmed patients under different conditions and to analyze the definition of positive Ct values under different conditions.

### 2.5 Statistical analysis

In this study, Excel was used for data summary and chart drawing, IBM SPSS Statistics (Version 21.0.0.0) was used for data statistical analysis. As for epidemiological parameters in this paper, we use five methods, namely, maximum likelihood estimation (MLE), moment matching estimation (MME), quantile matching estimation (QME), maximum goodness-of-fit estimation (MGE) and maximum spacing estimation (MSE) to fit seven common distributions such as Gamma distribution, Weibull distribution, Normal distribution, and Exponential distribution, and finally to arrive at the type of distribution applicable to the data, and R (Version 4.1.2) was used for fitting existing data and drawing.

## 3 Results

### 3.1 Natural history

**Fig. 1:**
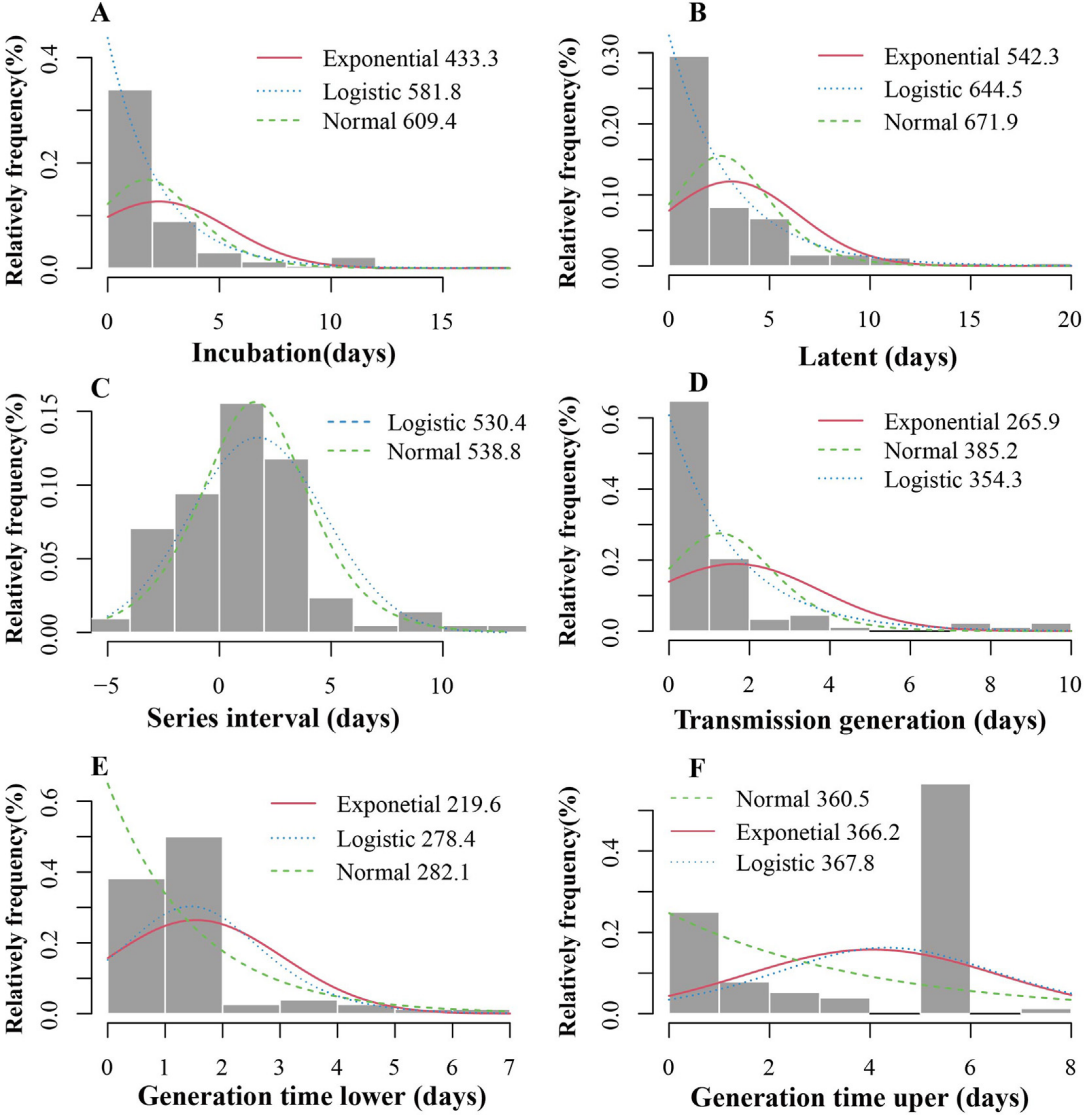
Fitting distribution of incubation period and intergenerational relationship. (A: Fitting distribution of incubation period; B: Fitting distribution of latent period; C: Fitting distribution of series interval; D: Fitting distribution of Transmission generation times; E: Fitting distribution of generation time lower; F: Fitting distribution of generation time uper.).

**Fig. 2:**
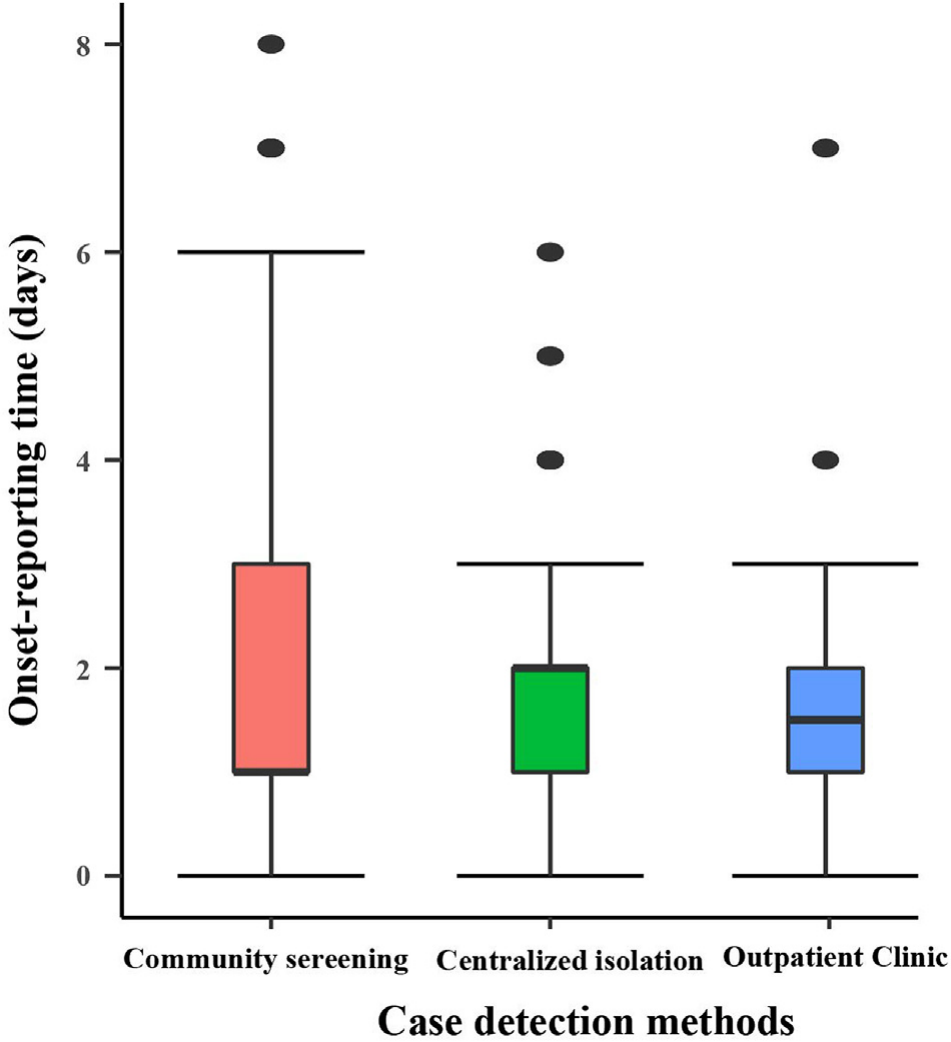
Difference in onset-reporting time of infected persons in different finding methods.

**Fig. 3:**
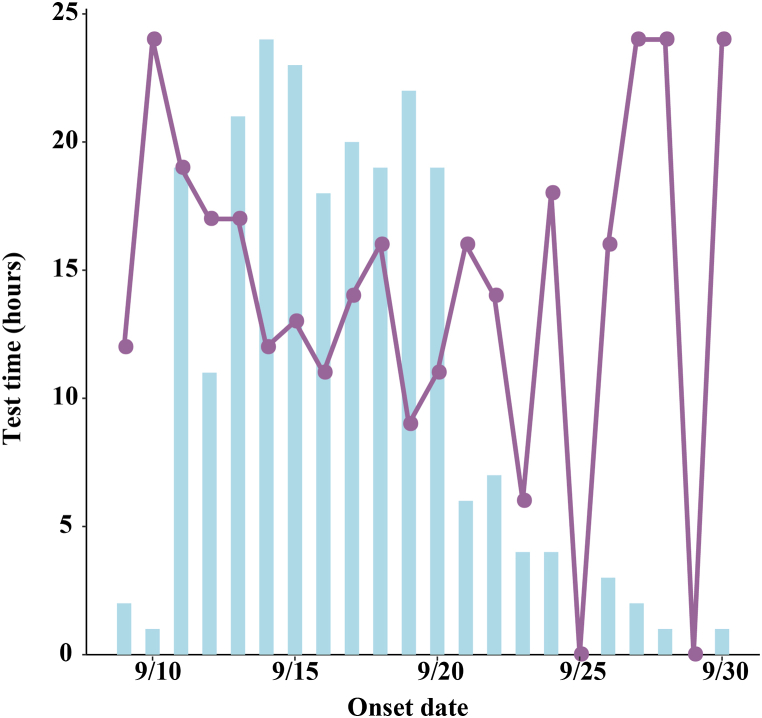
Time-dependent graph of the time required for detection.

The source of infection in Xiamen City was a case with a history of travelling to Xianyou County, Putian City, Fujian Province from September 5 to September 8. It can be assumed that this outbreak is an outflow of the outbreak in Putian City, Fujian Province. The incubation period of the cases was calculated to be 1–11 days, with a median of 4 days, summarization using MLE revealed that the incubation period was consistent with the Exponential (AIC = 433.3) distribution, Logistic (AIC = 581.8) distribution and Normal (AIC = 609.4) distribution, Fig. 1A. The latent period of the cases was calculated to be 0–20 days, with a median of 2 days, summarization using MLE revealed that the latent period was consistent with the Exponential (AIC = 542.3) distribution, Logistic (AIC = 644.5) distribution and Normal (AIC = 671.9) distribution, Fig. 1B. The onset-reporting time of confirmed cases varies according to the method of detection. As shown in Fig. 2, the median onset-reporting time for confirmed cases found in centralized isolation was 2 days (inter-quartile range [IQR]: 1–2 days). The median onset-reporting time for cases detected through community screening was 1 day (IQR: 1–3 days). The median onset-reporting time for cases identified through outpatient clinic was 1.5 days (IQR: 1–2.25 days). The median reporting-nucleic acid detection time for confirmed cases in the outbreak was 14.4 h (IQR:12–17 h). Based on the onset time, in the pre-epidemic period (September 9-September 19), with the continuous increase of confirmed cases, the diagnosis time required for PCR testing in Xiamen also decreased from 25 h to a minimum of 7 h, as detailed in Fig. 3.

### 3.2 Intergenerational relationships

We defined the first contact time of the second-generation cases minus the first contact time of the first-generation cases as the lower limit of GT; the last contact time of the second-generation cases minus the last contact time of the first-generation cases as the upper limit of GT, and calculated the interval of GT. The upper limit of GT was also calculated to be 4.03 ± 2.54 days, concentrated in 6 days, and the lower limit of GT was 1.54 ± 1.52 days, concentrated in 2 days. MLE was used to fit the distribution of the upper and lower limit of GT, and it was found that the uper limit of GT conformed to three distributions, namely: Exponential distribution (AIC = 366.2), Normal distribution (AIC = 360.5) and Logistic distribution (AIC = 367.8), the lower limit of GT conformed to three distributions, namely: Exponential distribution (AIC = 219.6), Normal distribution (AIC = 282.1) and Logistic distribution (AIC = 278.4) (Fig. 1E and F). After data processing and analysis, a total of 88 confirmed patients were included in the calculation of TG. As shown in Fig. 1D, the average TG for this outbreak was 1.67 ± 2.11 days, MLE was used to fit the distribution of TG, the results showed that the distribution of TG fit to Logistic distribution (AIC = 354.3), Normal distribution (AIC = 385.2) and Exponential distribution (AIC = 265.9). A total of 106 individuals were included in the calculation of SI, and the average SI in this case was 1.7 ± 3.0 days. MLE was also used to fit the distribution of SI. Unlike the previous two times, the distribution of SI fit to Logistic distribution (AIC = 530.4) and Normal distribution (AIC = 538.8), Fig. 1C.

### 3.3 Ct value

**Fig. 4:**
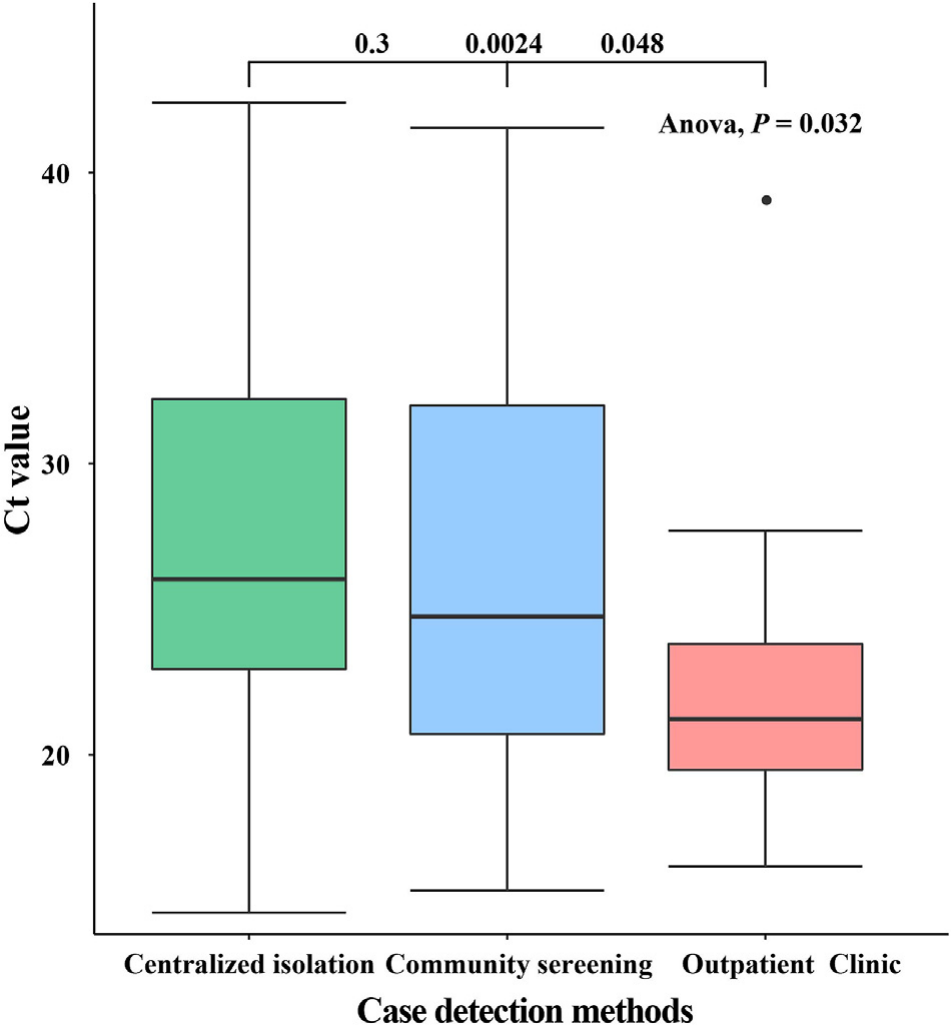
Differences in Ct values of infected persons with different detection methods. (Ct∗: cycle threshold).

**Fig. 5:**
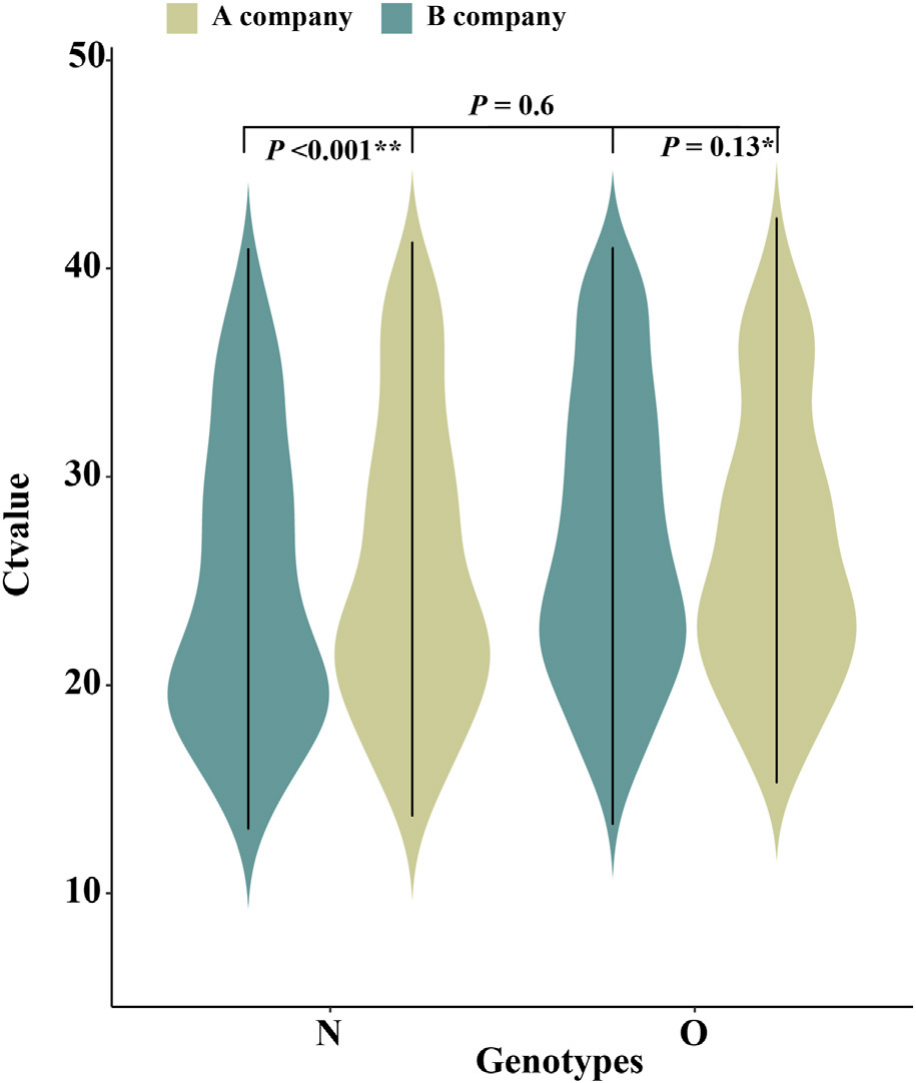
Differences in Ct values between different testing companies and genotypes. (Ct∗: cycle threshold).

**Fig. 6:**
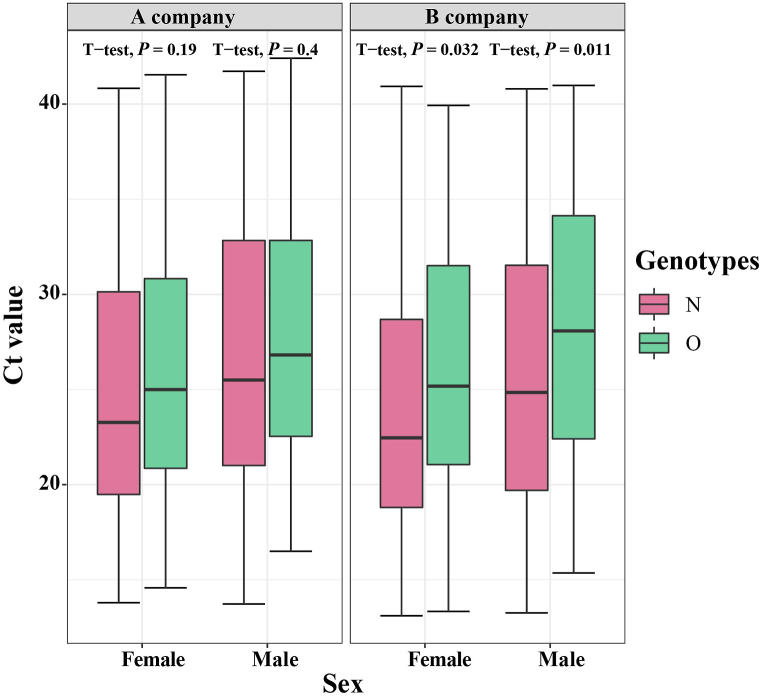
Gender differences in Ct values for different testing companies and genotype testing. (Ct∗: cycle threshold).

**Fig. 7:**
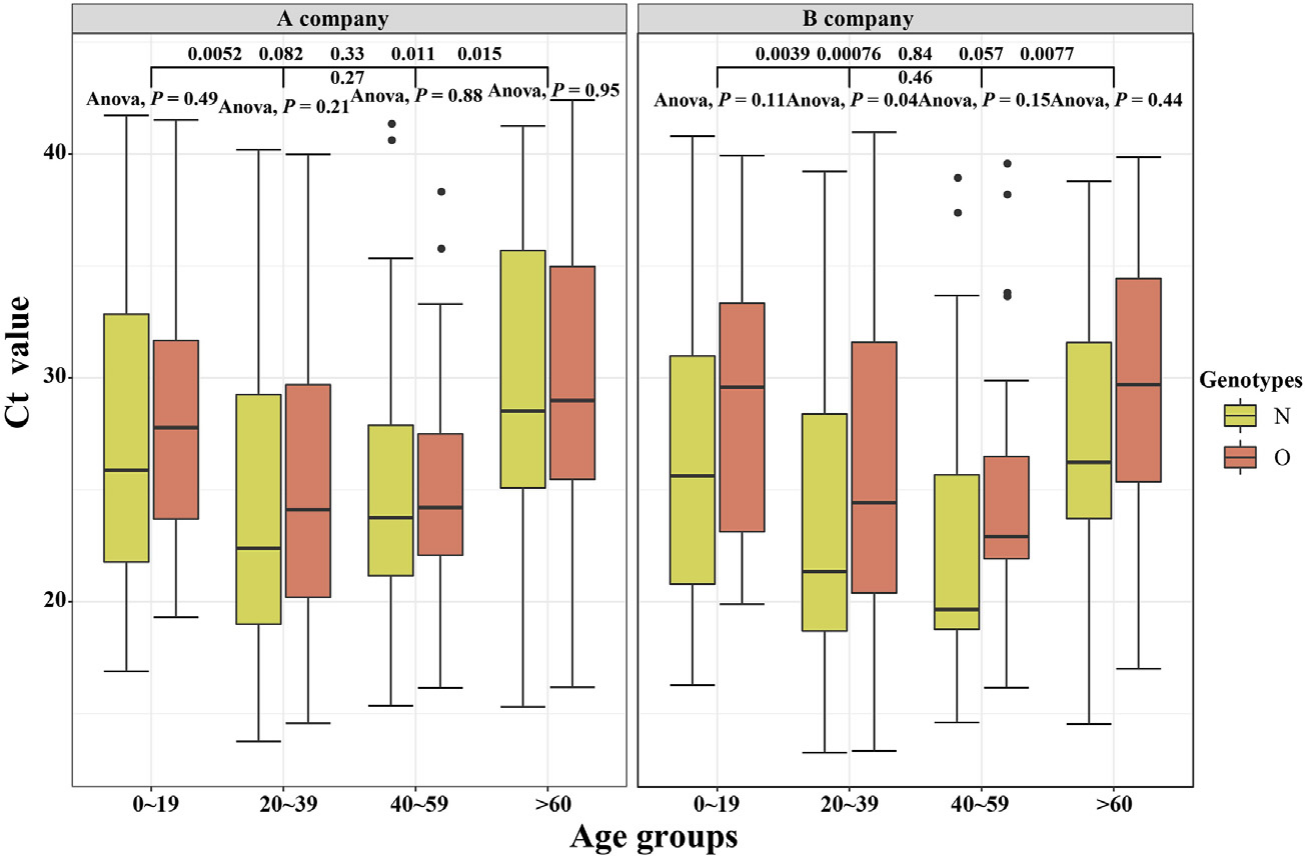
Age-specific differences in Ct values for different testing companies and genotype testing. (Ct∗: cycle threshold).

**Fig. 8:**
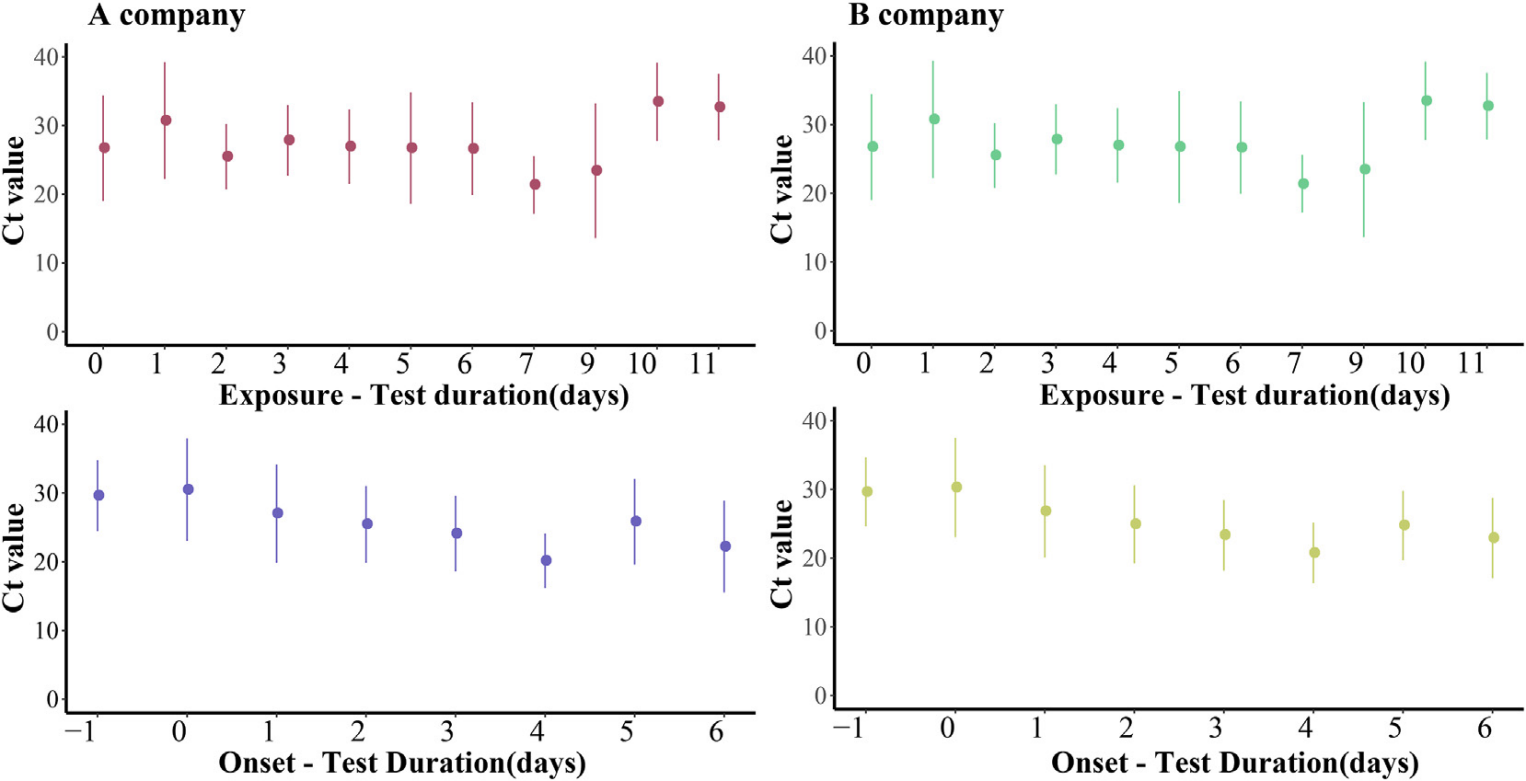
Differences in Ct values detected by two testing companies at different exposure-to-detection durations and onset-to-detection durations. (Ct∗: cycle threshold).

A total of 236 confirmed cases have been reported in this outbreak in Xiamen. After excluding one closed-loop manager, 59% of confirmed cases were detected in centralized isolation, 35% in community screening, and only 6% in outpatient clinic. As is shown in Fig. 4, The Ct values for patients identified in centralized isolation and community screening were much higher than those for confirmed patients with outpatient clinic, with a statistically significant difference (*P* < 0.05). The values were below 25. Two different brands of detection reagents, from A company and B company, were mainly used for nucleic acid detection, and both N and O genotypes were tested. The mean Ct values for the O genotype tested by A company were 26.8 ± 6.7, the mean Ct value of the N genotype were 25.8 ± 7.3, while the mean Ct values for the O genotype and the N genotype tested by B company were 27.2 ± 6.9 and 24.9 ± 7.1, respectively. We used *t*-test to analyze the Ct values of different reagents and different genotypes, and found that there was no statistical difference in the Ct values between different detection reagents (*P* = 0.6), but the Ct values of different genotypes were differences within and between B company and A company (*P* < 0.001, *P* < 0.13), as shown in Fig. 5. In Fig. 6, there was little difference in Ct values between males and females in the assay reagents, but males generally had higher Ct values than females. The Ct value of N genes was relatively lower than those of O genes, with most Ct values below 30, while the average Ct value of N genotype assays were above 25. The differences between the two genotypes in males and females were analyzed using *t*-test, and were found to be statistically significant (*P* < 0.05). Among different age groups, the average Ct values ranged from 25 to 30 in patients diagnosed at 0–19, from 23 to 27 in patients diagnosed at 20–39, and from 20 to 25 in patients diagnosed at 40–59. The average Ct value was between 20 and 25. The Ct value in the confirmed cases gradually decreased with age, but when the age was over 60 years old, the in vivo Ct values were higher, which were generally concentrated between 28 and 35. There were some differences in the in vivo Ct values in different age groups, as shown in Fig. 7. The differences in Ct values over the duration of exposure to assay were analyzed. When the duration of exposure to diagnosis was within one week, the in vivo Ct value were in a relatively stable state, with an average value between 20 and 30. After 7 days of exposure, Ct in vivo value reaches its lowest. The Ct values showed a decreasing trend with time during the time period from onset to detection, which is presented in Fig. 8.

**Fig. 9:**
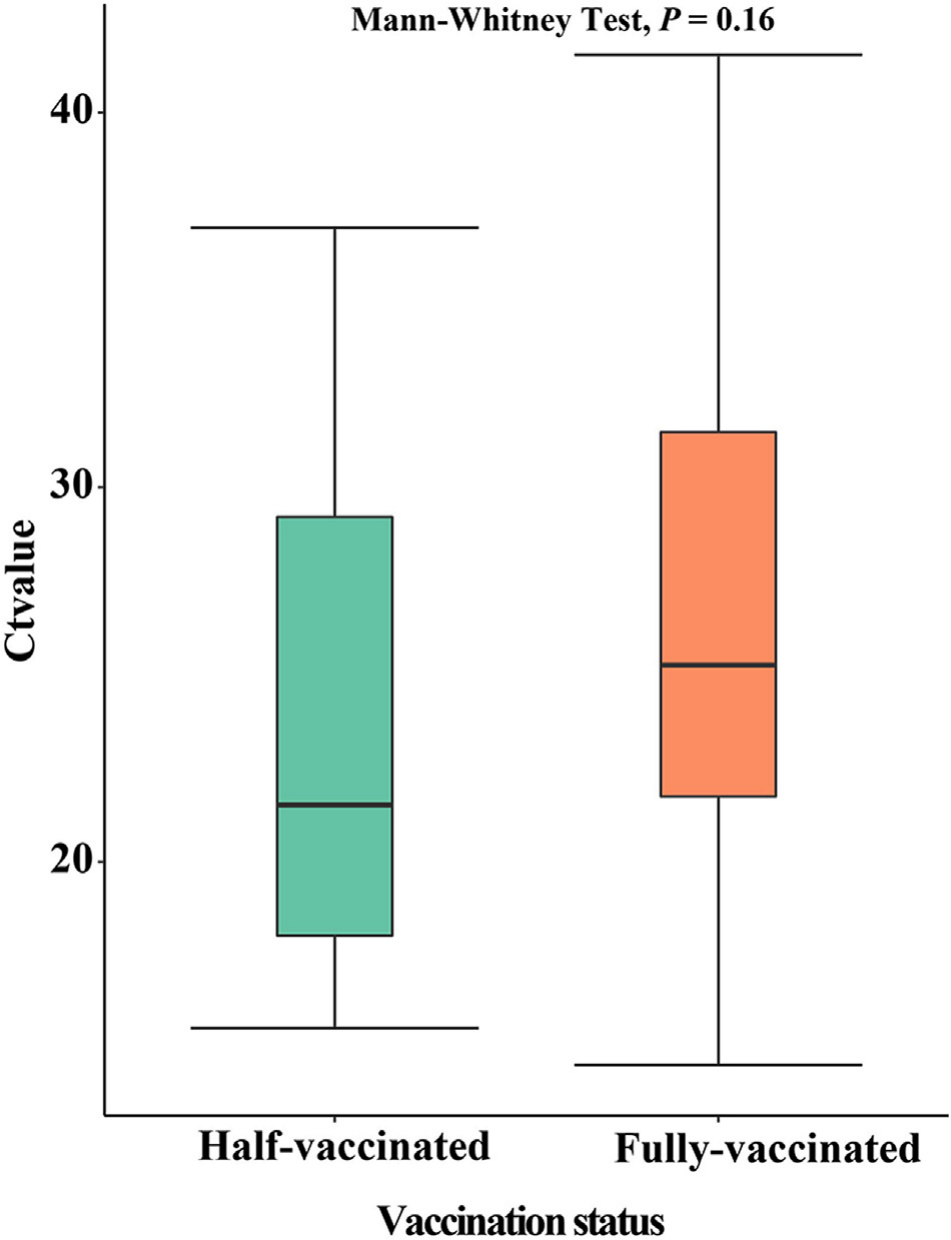
Differences of half-vaccination and vaccination in Ct value. (Ct∗: cycle threshold).

**Fig. 10:**
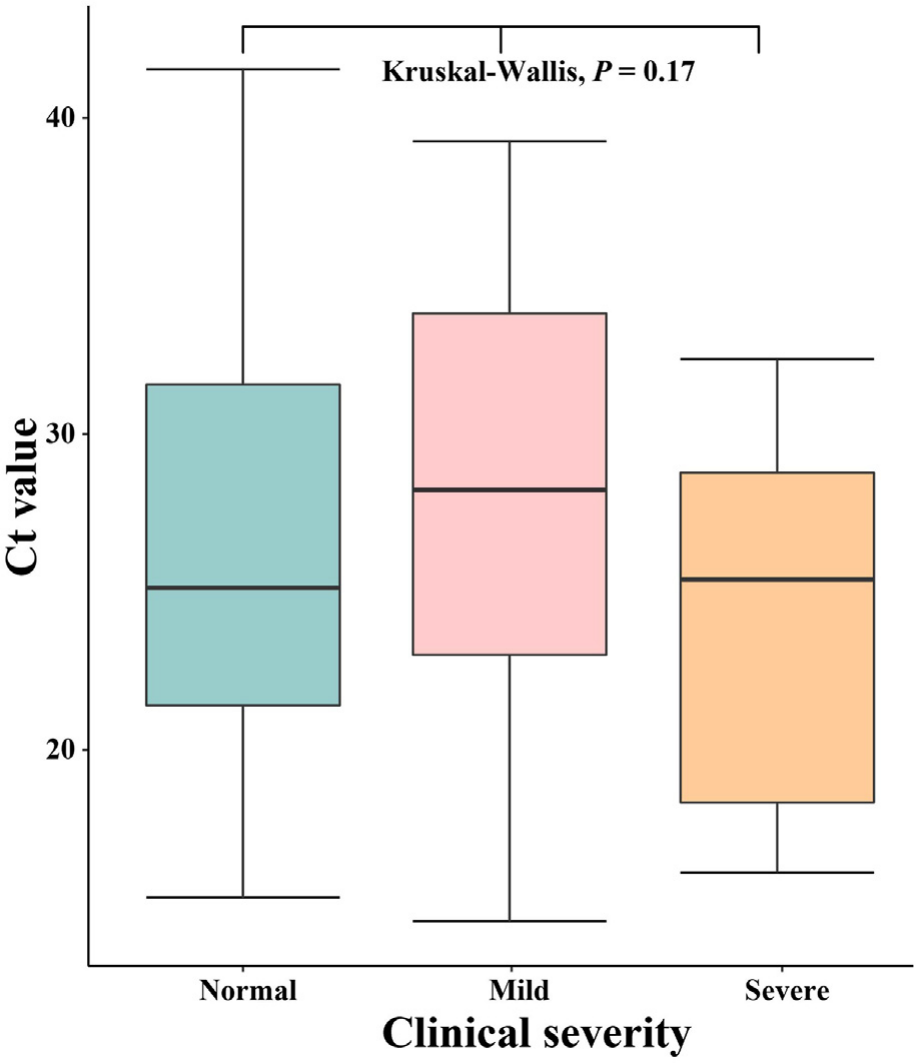
Differences in Ct values of infected patients with different clinical severity.

In this outbreak, most of the confirmed cases have completed the vaccination. For different vaccination situations, we use the *t*-test for comparative analysis. There was no significant difference in Ct values under different inoculation conditions (*P* = 0.16). The average Ct value of patients vaccinated in the whole course was around 25, and below 25 for half-vaccinated patients, Fig. 9. We explored whether there were differences in Ct values among patients with different severity. The data were analyzed using Mann-Whitney Test, and the average patient had a relatively average Ct value, concentrated between 23 and 31. There were no significant differences in the Ct values between patients with mild and severe symptoms (P > 0.05). Mild patients had higher Ct values, mostly above 27, while most of the severe patients had Ct values below 25. The difference in Ct value between different clinical severity has no statistically significant differences (P > 0.05), see Fig. 10 for details.

## 4 Discussion

In this study, we collected data on the first outbreak of Delta variant COVID-19 in a factory, analyzed the natural history parameters of Delta variant in environments where people congregate, such as incubation period and time to diagnosis, and we calculated the intergenerational relationship of Delta to estimate the transmissibility of Delta variant in specific sites. In addition, cycle thresholds for confirmed patients were analyzed to assess the differences in Ct values under different conditions and to provide a theoretical basis for diagnostic criteria and timing.

The first case of this outbreak had a history of sojourn in a COVID-19 infected area (Putian City, Fujian Province) from September 5 to September 8, 2021, while the specificity of his occupation (factory worker) led to the outbreak of this aggregated outbreak (Poole et al., 2021). The median incubation period calculated for confirmed patients in this study was 4 days, which is similar to the incubation period of the outbreaks of the Delta variant in other cities, but higher than the currently prevalent Omicron variant (Del Aguila-Mejia et al., 2022; Wang et al., 2021), this may be due to that the incubation period of the virus variant decreases with the variation of the virus (Liu et al., 2021). During the outbreak, positive patients were found in Xiamen mainly by three ways: community screening, active consultation and centralized isolation, and our study found that there was no statistical difference in the time interval between onset and reporting of patients in these three discovery methods, which should be combined to detect and dispose of cases as early as possible in the process of outbreak prevention and control (Aziz et al., 2020). In addition, we calculated that the median reporting-nucleic acid detection time for confirmed cases in the outbreak was 14.4 h (IQR:12–17 h), which is less than 3–10.4 days of that in another study (Faes et al., 2020), this can be attributed to the prevention and control measures taken by Xiamen, such as nucleic acid detection and centralized isolation. In terms of time to onset, diagnosis duration for confirmed cases continued to decrease in the early part of the outbreak (September 9-September 19), suggesting that during the outbreak, as the number of cases continues to increase, diagnosis duration could be continuously reduced by enhanced nucleic acid testing, centralized isolation and other measures. However, due to limited data, we were unable to assess the effectiveness of each intervention.

Therefore, we found that lower limit of GT was more in line with the exponential distribution (Exponential), which is similar to the results of another study. (Ganti et al., 2022). To calculate TG, it is necessary to clarify the time when two generations of cases become contagious. In this study, the mean time to TG in the epidemic was found to be 1.67 ± 2.11 days, which was more in line with the exponential distribution. The mean of TG of existing studies is 4.0 days (95%CI: 3.3–4.6), which is higher than our results (Zhao, 2020). TG is affected by specimen collection time, frequency, method and detection sensitivity of the test, therefore is more dependent on laboratory testing, and its value in field epidemiology still needs to be further explored. Since this outbreak was a cluster outbreak in factories, most of the cases were found at the centralized isolation points, and the time of positive tracing was more accurate. In this epidemiological investigation of the outbreak, the time of symptom onset of each infected person was relatively easy to obtain (Hong et al., 2020), so SI is a common indicator of intergenerational relationship in field epidemiology (Zhao, 2020). The mean intergenerational time for SI was calculated to be 1.7 ± 3.0 days, which is more in line with the Logistic distribution. The SI of this article is shorter than that of the pre-Delta variant (Li et al., 2020), which may be related to the fact that this outbreak is caused by Delta variant, and the virulence of Delta variant is stronger than pre-Delta variant, the symptoms of the disease will appear earlier, so the disease may be detected as soon as possible (Alene et al., 2021). The average TG time of this epidemic is 1.67 days, which was shorter than 4 days in other places, indicating that the transmission time of this epidemic was much shorter than in other areas. The clustered outbreak of factories caused by the Delta variant is caused by high human-to-human contact (Zhang et al., 2020). During the next outbreak prevention and control, we need to focus on places with a high concentration of people (Fowlkes et al., 2021). Meanwhile, the time difference between SI and GT is not significant. Even if the basic reproduction number of this epidemic is estimated according to SI, it can more accurately reflect the development trend and transmission intensity of this epidemic, and thus better prevention and control measures can be taken (Wallinga & Lipsitch, 2007).

Ct values as one of the diagnostic methods for COVID-19 were found to be significantly different in terms of genotype, age, occupation and interval between exposure and testing. From the perspective of testing reagents and genotypes, we tested for both N and O genotypes. The analysis revealed no statistical difference in Ct values between the testing reagents of different companies (P > 0.05), but not between genotypes. The variation within the same species of assay (P < 0.001, P < 0.05) also prompted us to be cautious when analyzing a single Ct value, as it may be related to the genes and assay products analyzed. Similar to some studies, Ct values were not significantly different in gender, but our study found that Ct values were generally higher in male patients than in females. We found that the mean Ct values for patients diagnosed in different age groups ranged from 25 to 30 for patients aged 0–19 years, 23–27 for patients aged 20–39 years, and 20–25 for patients aged 40–59 years. this also suggests that adults are more likely to be infected with COVID-19 than older adults and children, and need to focus on. This is also consistent with the results of our previous study (Zhao et al., 2020). Based on previous studies, this may be related to physiological characteristics, presence or absence of vaccination, timing of sampling and exposure patterns at different ages (Maltezou et al., 2020; Zhao et al., 2020). Also, we found some differences in Ct values among patients with different occupations (p < 0.05). The Ct values would be relatively high in the population of children in nurseries, self-employed population, retired, unemployed, and waiters. It has also been reported that the transmission of the virus is stronger in populations where aggregation often occurs in occupational settings. Our study found that Ct values (mean values below 30) showed fluctuating changes after exposure. Trends in Ct values were similar to those simulated by an agent-based model of one study (Aranha et al., 2021; Quilty et al., 2021). When the time interval from exposure to diagnosis is within one week, the in vivo Ct values are relatively stable with a mean value of 20–30.7 days after exposure, the in vivo Ct values drop to the lowest level. In terms of vaccination, Ct values are higher in unvaccinated patients than in vaccinated patients, but Ct values are lower in patients who take one dose of vaccine than in patients with two doses, suggesting that completion of vaccination reduces transmission of novel coronaviruses, but further validation is needed to verify that transmission is greater in patients who are vaccinated halfway (Hsu et al., 2021). It has been shown that the Ct values in reported cases are lower than 40 (Arons et al., 2020; WHO), which is also the same as our results, and it may range from 23 to 31 in patients with the common type, but are higher in mild patients, mostly above 27, and in severe patients, mostly below 25 (Zheng et al., 2020). In addition, the Ct values of patients diagnosed in different testing methods varies. Patients identified by centralized isolation and community screening had Ct values between 23 and 32, which were higher than those of confirmed patients who actively sought medical care (P < 0.05). Patients who were actively seeking medical care all had Ct values below 25, and they were in the clinical symptomatic stage at the time of outpatient clinic. At this time, the "detoxification" capacity was relatively significant and the viral load was higher than in the other two groups. Therefore, we recommend symptomatic patients to visit the fever clinic promptly. Seeking medical attention to improve the ability to detect cases early, so as to cut off the transmission chain in time and control the epidemic.

## 5 Conclusion

The outbreak of COVID-19 in Xiamen was caused by a factory cluster, and the incubation period of this virus is very short. Active medical treatment, nucleic acid screening and centralized isolation can improve the ability to detect cases, shorten the time between case occurrence and diagnosis, and help control the outbreak as soon as possible. In the process of outbreak transmission assessment, symptom intergenerational relationship (SI) is often used instead of infection intergenerational relationship (TG) to calculate the effective reproduction number and real-time reproduction number, which can also reflect the developing trend and transmission intensity of the outbreak. In addition, the analysis of Ct values, combined with the individual characteristics of confirmed patients, can improve the positive detection rate and achieve targeted treatment.

## Authors' contributions

Tianmu Chen, Bin Deng, Weikang Liu, Zhinan Guo, Li Luo and Chenghao Su designed research; Bin Deng, Weikang Liu, Zhinan Guo, Li Luo, Tianlong Yang, Jiefeng Huang, Buasiyamu Abudunaibi, Xue Ouyang and Demeng Wang analyzed data; Tianmu Chen, Chenghao Su, Bin Deng, Zhinan Guo, Weikang Liu, Li Luo, Buasiyamu Abudunaibi, Tianlong Yang and Jiefeng Huang conducted the research and analyzed the results; Tianmu Chen, Chenghao Su, Bin Deng, Weikang Liu, Zhinan Guo, Li Luo, and Buasiyamu Abudunaibi wrote the manuscript. All authors read and approved the final manuscript.

## Funding

This work was partly supported by the 10.13039/100000865Bill & Melinda Gates Foundation (INV-005834) and the 10.13039/501100012166National Key Research and Development Program of China (2021YFC2301604) and the 10.13039/501100005270Fujian Provincial Department of Science and Technology pilot project (2020D019).

## Availability of data and materials

Data supporting the conclusions of this article are included within the article.

## Ethics approval and consent to participate

COVID-19 control and prevention are part of CDC's routine responsibility in Xiamen City, China. Therefore, this study does not need institutional review and informed consent. All data analyzed data are anonymous.

## Declaration of competing interest

The authors declare that they have no known competing financial interests or personal relationships that could have appeared to influence the work reported in this paper.
